# Calcium Silicate-Activated Gelatin Methacrylate Hydrogel for Accelerating Human Dermal Fibroblast Proliferation and Differentiation

**DOI:** 10.3390/polym13010070

**Published:** 2020-12-27

**Authors:** Fong-Sian Lin, Jian-Jr Lee, Alvin Kai-Xing Lee, Chia-Che Ho, Yen-Ting Liu, Ming-You Shie

**Affiliations:** 1 x-Dimension Center for Medical Research and Translation, China Medical University Hospital, Taichung City 40447, Taiwan; lfs1982@gmail.com (F.-S.L.); Leekaixingalvin@gmail.com (A.K.-X.L.); u102001416@cmu.edu.tw (Y.-T.L.); 2 School of Medicine, China Medical University, Taichung City 40447, Taiwan; D33977@mail.cmuh.org.tw; 3 Department of Plastic & Reconstruction Surgery, China Medical University Hospital, Taichung City 40447, Taiwan; 4 Department of Bioinformatics and Medical Engineering, Asia University, Taichung City 41354, Taiwan; sethho@asia.edu.tw; 5 3D Printing Medical Research Institute, Asia University, Taichung City 41354, Taiwan; 6 School of Dentistry, China Medical University, Taichung City 40447, Taiwan

**Keywords:** gelatin–methacryloyl, fibroblast, hydrogel, calcium silicate, extracellular matrix remodeling

## Abstract

Wound healing is a complex process that requires specific interactions between multiple cells such as fibroblasts, mesenchymal, endothelial, and neural stem cells. Recent studies have shown that calcium silicate (CS)-based biomaterials can enhance the secretion of growth factors from fibroblasts, which further increased wound healing and skin regeneration. In addition, gelatin methacrylate (GelMa) is a compatible biomaterial that is commonly used in tissue engineering. However, it has low mechanical properties, thus restricting its fullest potential for clinical applications. In this study, we infused Si ions into GelMa hydrogel and assessed for its feasibility for skin regeneration applications by observing for its influences on human dermal fibroblasts (hDF). Initial studies showed that Si could be successfully incorporated into GelMa, and printability was not affected. The degradability of Si-GelMa was approximately 20% slower than GelMa hydrogels, thus allowing for better wound healing and regeneration. Furthermore, Si-GelMa enhanced cellular adhesion and proliferation, therefore leading to the increased secretion of collagen I other important extracellular matrix (ECM) remodeling-related proteins including Ki67, MMP9, and decorin. This study showed that the Si-GelMa hydrogels were able to enhance the activity of hDF due to the gradual release of Si ions, thus making it a potential candidate for future skin regeneration clinical applications.

## 1. Introduction

The human skin is responsible for maintaining homeostasis and has a huge role to play in regulation wound healing and regeneration. Wound healing is a complex process that requires specific interactions between multiple cells such as fibroblasts, mesenchymal, endothelial, and neural stem cells [1]. Wound healing is known to involve four major phases: the hemostasis phase, inflammatory phase, proliferative phase, and remodeling phase. Fibroblasts are known to be a critical component of wound healing because they are involved in secreting proteins such as proteoglycan and fibronectin that are required for building extracellular matrix of tissues. In addition, fibroblasts also secrete angiogenic factors such as basic fibroblast growth factor (bFGF) and vascular endothelial growth factor (VEGF), both of which are known to influence and upregulate neovascularization and improve wound healing [2]. Even though there is ample knowledge regarding the regeneration of simple wounds, the healing of extensive and deep wounds still remains as a hurdle for doctors and scientists alike. Since the last decade, scientists have been attempting to find some methods to accelerate wound healing [3]. Current solutions to covering wounds include autografts, allografts, and xenografts. However, each method has its own limitations that greatly restrict its application in the area of wound healing [4]. Therefore, many researchers have attempted to fabricate or modify natural biomaterials in order to fabricate tissue engineered substitutions for our native skin [5]. The main aim of tissue grafts and tissue engineered substitutions is to support the healing of epidermis and the dermis in large-scale wounds. Recent advances in biomedicine and materials science have enabled the implementation of strategies in fabricating unique scaffolds to imitate the biological, architectural, and functional characteristics of native skin. In fact, there were many synthetic biomaterials available provided they are clinically used, but they could not provide a supportive environment for tissue regeneration in severely injured skin [6].

Hydrogels are polymeric biomaterials with three-dimensional cross-linked internal architectures that can be used as a barrier for wounds [7]. Hydrogels have high customizability that make it possible to personalize their different characteristics for different settings [7,8]. Studies have shown that hydrogels with a higher degree of adhesiveness to tissues enhance tissue regeneration by bringing tissues together and also prevent the formation of scars due to traditional suturing methods [9,10]. Moreover, the hydrogels made from gelatin have a dynamics macromolecular network and biomimic microenvironment that has a high-water content [11,12]. Gelatin is a well-known biomaterial that is commonly used in the fabrication of hydrogels in the form of a gelatin derived from collagen, thus making it possible to retain the natural motifs of the extracellular matrix and providing excellent biocompatibility, biodegradability, and non-toxicity [13,14]. Furthermore, gelatin can be modified with methacryloyl groups (GelMa) to allow photo-crosslinking so as to provide customizable mechanical properties [15]. The mechanical strength of GelMa could be adjusted by varying the concentration of methacrylol, strength of UV photo-crosslinking, and duration of UV exposure. However, there is a safety threshold for the above parameters as too high a concentration of methacrylol and UV is known to be cytotoxic to cells. Therefore, there is a limit to the mechanical strength of GelMa that we can control; thus, there is a need for us to come up with innovations to overcome this bottleneck [16,17,18,19].

Bioglass (BG) and calcium silicate (CS) materials are inorganic biomaterials that have been shown to enhance vascularization and tissue regeneration through the release of silicon (Si) ions [20,21,22]. Furthermore, CS-based materials had been proved to be able to stimulate the angiogenesis of endothelial cells by inducing the secretion of angiogenic-related growth factors such as VEGF and bFGF from fibroblasts [23,24]. This is due in part to the sustained release of Si and Ca ions into its surrounding fluids after coming into contact with body fluids [22]. The growth factors not only contribute to the attraction between endothelial cell and fibroblasts but also enhance the secretion of extracellular matrix (ECM)-related proteins from these cells [25]. Furthermore, it had been previously reported that BG can release calcium ions gradually, which may contribute to the better adhesion of hydrogels with native tissues in subsequent chelation reactions [26].

In this study, we developed a GelMa solution containing Si ions by applying the extract from calcium silicate to allow the dissolution of GelMa. Then, the GelMa hydrogels were further transformed into stable cross-linked hydrogel disks by exposing them to UV light. To the best of our knowledge, there have been no such combinations of hydrogels used to assess wound-healing capabilities. Thus, the mechanical and biological properties of the various concentrations were investigated. In addition, animal models were used to assess the wound healing, angiogenesis, and collagen deposition capabilities of the proposed Si ion hydrogels. From this study, it was found that a GelMa hydrogel containing Si ions has potential as an alternative to current wound-healing strategies.

## 2. Materials and Methods

### 2.1. Preparation of the Extracts of CS Powders

CS were prepared as according to our previous published methods [27]. Commercially proven, analytically graded reagents were purchased from Sigma-Aldrich (Sigma-Aldrich, St. Louis, MO, USA). First, 70% calcium oxide (CaO) was mixed with 25% silicon dioxide (SiO_2_) and 5% alumina oxide (Al_2_O_3_) and subsequently sintered at 1400 °C for 2 h and cooled to room temperature. The mixtures were placed into 99.5% ethanol and further ground with agate milling balls in a planetary ball mill machine (Retsch PM-100, Retsch GmbH, Germany) for 12 h. Then, the mixture was dried at 100 °C in an oven for 12 h. In addition, extracts of the CS powders were obtained following the revised version of the International Standard Organization (ISO/EN 10993-5). Briefly, a steam sterilization method was utilized to sterilize the CS powders before soaking in a tris-buffer (25 mM, pH = 8.5). After stirring for 24 h, the mixtures were filtered, and the supernatants were sterilized using a 0.2 μm filter. The Si concentration in the extracts were analyzed using inductively coupled plasma atomic emission spectrometry (ICP-AES; Perkin-Elmer OPT 1MA 3000DV, Shelton, CT, USA).

### 2.2. Preparation of Si-Contained GelMa Hydrogels

Type B-based gelatin methacrylate commercial powder (GelMa, Ever Young BioDimension, Taichung, Taiwan) was used in this study. The GelMa was first dissolved in CS extracts with varying concentrations of Si ions (Si0: 0 mM, Si0.5: 0.5 mM and Si1.0: 1.0 mM) and 0.5% *w*/*w* of Irgacure 2959 photo-initiator (Sigma-Aldrich) to allow photo-curing. Pluronic^®^ F-127 (F127, Sigma-Aldrich) was extruded via an extrusion-based BioX bioprinter (Cellink, Gothenburg, Sweden) before the hydrogels were deposited into the molds. Then, the F-127 and GelMa hydrogels were exposed to 10 mW/cm^2^ 365 nm UV (Spot Cure Series, SP11, Ushio, Japan) at a distance of 30 cm for 90 sec. To obtain the GelMa hydrogels, the F-127/GelMa hydrogel was immersed in cold water to allow dissolution of the F-127 support. 

### 2.3. Si-GelMa Hydrogel Characterizations

Attenuated total reflectance−Fourier transform infrared spectroscopy (ATR-FTIR, Bomem DA8.3, Hartman & Braun, Brantford, ON, Canada) was utilized to investigate the chemical structure and the possible organic–inorganic interactions in the hydrogel specimens in a wavenumber range from 4000 to 500 cm^−1^. In addition, the mechanical properties of the GelMa hydrogels were measured using tensile tests conducted with the EZ-Test machine (Shimadzu, Kyoto, Japan). The specimens were printed into the shape of a dumbbell with a thickness of 2 mm. Similarly, F127 was used as the support structure for the GelMa. For the test, a tensile pull at a constant rate of 1 mm/min was applied to the hydrogel until the hydrogel tore completely, and the corresponding stress and strain were then recorded upon the complete lesion of the hydrogels. The specimens used in the above analysis were tested in triplicate. In addition, the Young’s modulus was used to calculate the crosslink densities, which represents the mole number of active chain segments per unit volume (m^3^) of each hydrogel group by using the rubber elasticity theory [28] according to the following formula:

$$\mathrm{Crosslink}\;\mathrm{Density}=\frac{E}{3\mathrm{RT}}$$

where *E*, R, and T stand for the Young’s modulus in Pascal (Pa), universal gas constant in (8.3144 J/mol·K), and absolute temperature (K), respectively. In addition, the microstructure of the hydrogel was investigated under a cryo-scanning electron microscope (cryo-SEM, JSM-7800F, JEOL, Tokyo, Japan).

### 2.4. In Vitro Immersion Study and Weight Loss

The in vitro immersion test was applied to evaluate for the biodegradation properties of the Si-GelMa hydrogel. The hydrogels were immersed in a 50-mL centrifuge tube containing 40 mL of simulated body fluid (SBF) at 37 °C. The ionic composition of the SBF in this study comprised 7.9949 g of NaCl, 0.3528 g of NaHCO_3_, 0.2235 g of KCl, 0.147 g of K_2_HPO_4_, 0.305 g of MgCl_2_•6H_2_O, 0.2775 g of CaCl_2_, and 0.071 g of Na_2_SO_4_ in 1000 mL of distilled water. In addition, hydrochloric acid and tris base ((CH_2_OH)_3_CNH_2_) were used to buffer the solution to pH 7.4. Then, after immersion, the Si-GelMa hydrogels were removed from the SBF and assessed for their weight. In addition, the profile of the released Ca, Si, and P ions was also analyzed at different time points using ICP-AES.

### 2.5. Cell Proliferation and Morphology

Human dermal fibroblasts (hDF; ScienCell Research Laboratories, Carlsbad, CA, USA) were used in this study for the subsequent in vitro studies. The hDF were cultured in commercial fibroblast medium (ScienCell Research Laboratories, Carlsbad, CA, USA) with the respective recommended antibiotics. The cells were trypsinized using trypsin/EDTA, collected using a hemocytometer, and seeded on Si-GelMa hydrogel at a density of 5 × 105 cells/mL. Next, PrestoBlue^®^ assay (Invitrogen, Grand Island, NY, USA) was used to assess the cell viability at specific culture durations. In brief, the medium was removed from the wells, followed by the use of phosphate buffered saline (PBS) to rinse the cells. Subsequently, fresh medium, mixed with PrestoBlue at a ratio of 9:1, was added to each well and incubated for 20 min at 37 °C. Then, the medium was transferred to a new 96-well plate. The detection of optical density (OD) was done at 570 nm with a reference of 600 nm using a spectrophotometer (TECAN Infinite Pro M200). The absorbance of cells without scaffolds were used as a control group (Ctl). The specimens were tested in triplicate from three independent experiments for each group. 

The cellular morphology of hDF was observed based on the F-actin cytoskeleton using nuclei staining. After the various culturing time points, the cells were washed with PBS and fixed in a 4% paraformaldehyde (Sigma-Aldrich) solution at room temperature for 20 min. Next, the cells were permeabilized in PBS containing 0.1 Triton X-100 (Sigma-Aldrich). The F-actin cytoskeleton was stained with the fluorescent dye (Alexa Fluor 488, Invitrogen, Carlsbad, CA, USA) conjugated with phalloidin according to the manufacturer’s instructions. In addition, 300 nM of 4’,6-diamidino-2-phenylindole (DAPI, Invitrogen) was used to stain the cell nuclei. Then, the cell morphology and cell distribution were observed using a confocal microscope (Leica Microsystems GmbH, Wetzlar, Hessen, Germany).

### 2.6. Western Blot Analysis

The hDF samples were seeded on the Si-GelMa hydrogels for different time points and washed three times with PBS. Then, the cells were lysed with an NP40 buffer (Invitrogen) to assess the protein concentrations using a bicinchoninic acid protein assay kit (Invitrogen). SDS-PAGE was used to separate the cell lysates (40 μg protein) according to the manufacturer’s instructions. They were subsequently transferred onto polyvinylidene difluoride membranes. Target primary antibodies (phospo-extracellular signal-regulated kinases 1/2 (pERK1/2), extracellular signal-regulated kinases 1/2 (ERK1/2), phospo-p38, p38, β-actin, Col I, Ki67, MMP9 and Decorin (Abcam) were placed onto the membranes and incubated overnight. Then, the membranes were washed and incubated with either horseradish peroxidase-conjugated anti-rabbit IgG (1:2000 dilution; Genetex, Hsinchu, Taiwan) or horseradish peroxidase-conjugated anti-mouse IgG (1:2000 dilution; Genetex) for 1 h at room temperature. Then, the Fusion-Solo chemiluminescence system (Vilber, Paris, France) and ECL detection reagents (Thermo Fisher, Waltham, MA, USA) were used to detect the signals emitted from the samples.

### 2.7. Collagen I Secretion

The hDF samples were cultured on the Si-GelMa hydrogels for 3 days and lysed with NP40 buffer to analyze the Col I expression via a Western blot. Target primary Col I antibody was purchased from Abcam. In addition, the hDFs were grown on different Si-GelMa hydrogels for 3 and 7 days. The hydrogels were rinsed using cold PBS and subsequently fixed by placing them in 4% paraformaldehyde (Sigma-Aldrich) for 20 min at normal room temperature. After this, the cells were permeabilized using 0.1% Triton in PBS, immune-stained with anti-Col I (Abcam), followed by anti-rabbit conjugated tetramethylrhodamine (TRITC, Invitrogen). The phalloidin affixed with Alexa Fluor 488 (1:300 dilution in PBS, Invitrogen) was used to stain the intracellular F-actin filaments. The immunofluorescence staining results for the hDFs on the hydrogels were obtained through observing under a Leica TCS SP8 X white light laser confocal microscope (Leica Microsystems GmbH, Wetzlar, Hessen, Germany).

### 2.8. Statistical Analyses

A one-way statistical analysis of variance (ANOVA) was performed to analyze the significance in the differences between the different experimental groups in each experiment. Scheffe’s multiple comparison test was used to determine the significant deviations for each sample. A *p*-value <0.05 was considered to be statistically significant, as indicated by “*”.

## 3. Results and Discussion

### 3.1. Characterization of Si-GelMa Hydrogel

**Figure 1:**
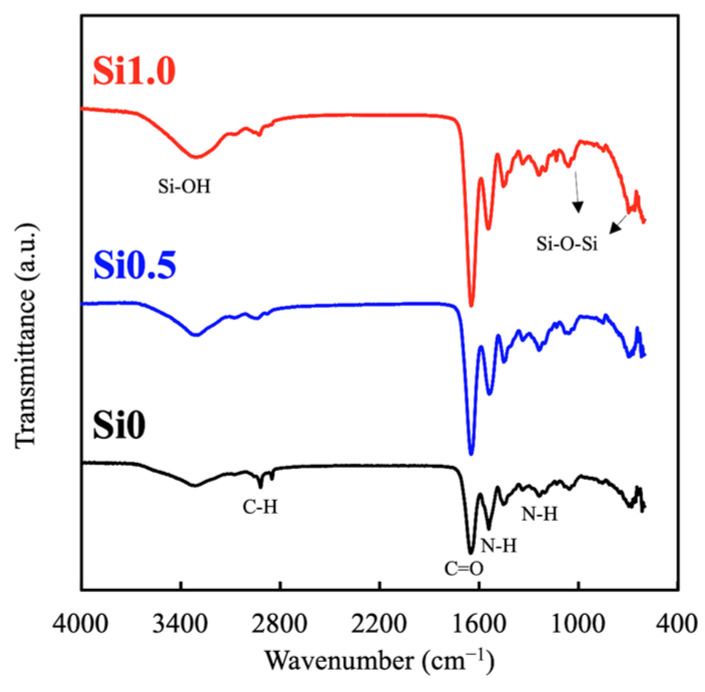
FTIR spectra of the various Si-GelMa (gelatin methacrylate) hydrogels.

ATR-FTIR was used to characterize and determine the presence of the specific Si and GelMa chemical groups in the hydrogel in order to study the interactions among the blended compounds. The results of the FTIR analysis are shown in Figure 1. As shown, Si1.0 had stronger peaks at 3400, 2790, 1650, 1550, and 110 cm^−1^, which represented Si–OH, C–H, C=O, N–H, and Si–O–Si, respectively. This result clearly indicated that Si could be incorporated successfully into the GelMa without affecting its structural integrity. Furthermore, different concentrations of Si ions could be loaded into the GelMa, as seen from the difference in intensity between Si1.0 and Si0.5. This is an important factor to note, because the goal was to retain the initial superior characteristics of the GelMa and yet add on the benefits of Si ions. Furthermore, the presence of Si-OH was reported to enhance the mechanical properties of hydrogels by supplying additional covalent bonds between gelatin [29]. 

**Figure 2:**
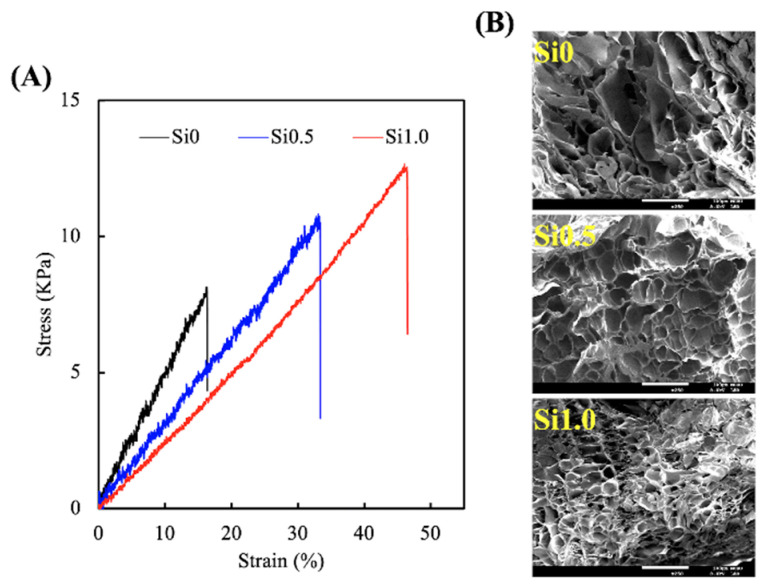
(**A**) Tensile stress–strain curves and (**B**) microstructure of the various Si-GelMa hydrogels.

To determine the mechanical properties of the various Si-GelMa hydrogels, an elastic modulus test was performed on dumbbell-shaped specimens exposed to 90 s of UV curing. The results of the tensile stress–strain curves are shown in Figure 2A. As expected, the stress–strain curves demonstrated a positive correlation between the various Si concentrations, with Si1.0 having a mechanical strength/elastic modulus of 12.4 kPa/0.26 MPa and Si0 having a mechanical strength/elastic modulus of 7.9 kPa/0.47 MPa. Si0 showed a typical brittle mechanical response, which was indicated by a sharp peak followed by a rapid decrease because the structure was unable to support the load. The addition of Si ions enhanced the peak mechanical strength of hydrogels by at least 20–60%; thus, it was hypothesized that the presence of Si-OH bonds was favorable to enhance and support the growth of skin tissue regeneration. In addition, the elastic modulus of Si1.0 hydrogel is similar to that of skin, thereby enabling optimal conformal skin–substitutes contact, adhesion, and transpiration [30]. Trappmann et al. reported that higher elastic moduli were favorable to the growth of keratinocytes. In their study, it was reported that keratinocytes cultured on 20% polydimethylsiloxane (PDMS, 2 MPa) had higher EGFR activation and proliferation as compared to keratinocytes cultured on 2% PDMS (180 kPa). Thus, it was hypothesized that Si ion-contained GelMa enhance and support skin tissue regeneration [31]. Ideally, the suitable mechanical properties allow for better surgical handling and adaptation to mechanical stress during healing [32]. The robust, yet tunable, mechanical properties of such hydrogels make them even more suitable for skin regeneration, since their characteristics can be personalized to suit the needs of an individual. In addition, we hypothesized that it was due to the higher cross-linking densities of Si1.0. To prove this point, the cryo-SEM images shown in Si1.0 had denser internal architectures with smaller pores, thus indicating that there was higher cross-linking in groups with higher Si concentrations (Figure 2B). Moreover, the calculated crosslink density of Si0, Si0.5, and Si1.0 were 67.1 ± 1.9, 47.1 ± 2.1, and 35.0 ± 2.9 mol/m^3^, respectively.

### 3.2. Degradation and Ion Released Properties of Si-GelMa Hydrogel

**Figure 3:**
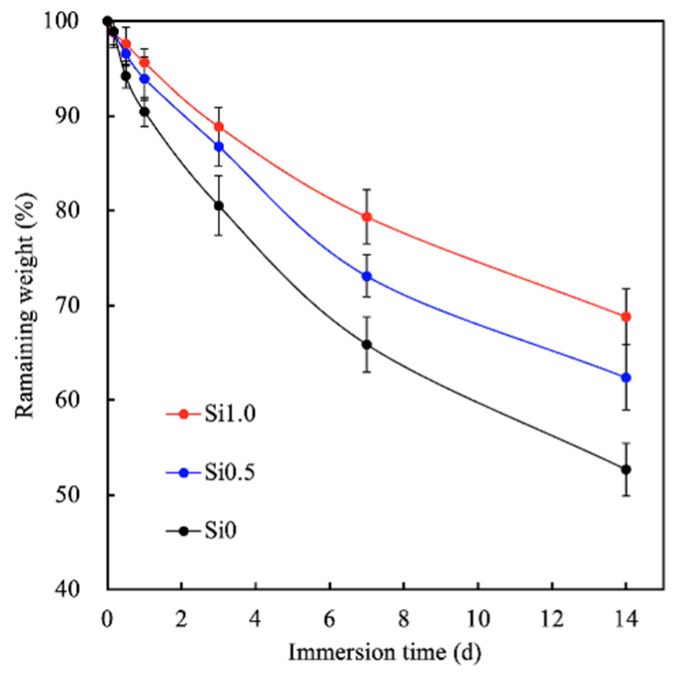
Degradation rates of the various Si-GelMa hydrogels after different durations of immersion.

To evaluate degradation, the various Si-GelMa hydrogels were immersed in SBF. The results are shown in Figure 3. As can be seen, the degradation rate decreased with increases in the Si concentration, with Si0, Si0.5, and Si1.0 having 53%, 65%, and 72% of their mass remaining after 14 days of incubation. Interestingly, our previous studies reported that pure GelMa hydrogels at concentrations of 5–15% exhibited almost complete degradation after 14 days of incubation [15]. Compared to pure GelMa hydrogens, Si ion-containing hydrogels are thought to be better suited for long-term wound healing because they can last until optimal wound healing occurs and prevent secondary infections and moisture loss [20]. 

**Figure 4:**
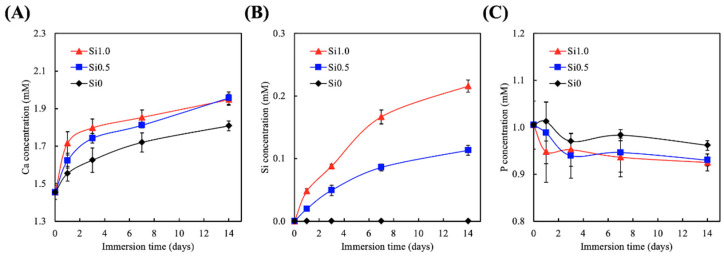
(**A**) Ca, (**B**) Si, and (**C**) P ions release analysis of hydrogels after immersion in simulated body fluid (SBF) for 14 days.

Various studies have tested and proven the bioactivity of CS-based materials and have shown that the gradual release of Si ions plays a role in regulating their bioactivity [33]. However, there has not been an emphasis on ion release for hydrogels incorporating Si. The various hydrogels were immersed in SBF for 14 days, and the concentrations of the Ca, Si, and P ions in the fluid were measured, as shown in Figure 4. As can be observed, the amount of released Ca and Si increased over the immersion period, and the amount of released P declined over time. The trend seen here was similar to the results published by Yu et al., who similarly fabricated Si-incorporated GelMa and tested its potential in regeneration of tissue defects [34]. In addition, Gao et al. fabricated a dual Si-incorporated sodium alginate hydrogel and showed that this hydrogel could promote in vivo wound healing as compared to a placebo [26]. It was shown in their study that the rapid ion exchanges on the surfaces of Si-GelMa hydrogels make the micro-environment alkaline, thus enhancing tissue–hydrogel interaction and integration. Therefore, we applied the CS-extract function in our hydrogel matrix design, in which the CS extract slowly and continues released Ca and Si ions to form a mildly alkaline environment in support of the amide-forming reaction. In addition, the adhesion behavior between the hydrogel and tissue was further promoted by the release of Ca ions, which were shown to chelate with the carboxyl group on tissues. It has been reported that the presence of Si ions in a wound area promotes angiogenesis and wound healing [26].

### 3.3. Cell Adhesion and Proliferation

**Figure 5:**
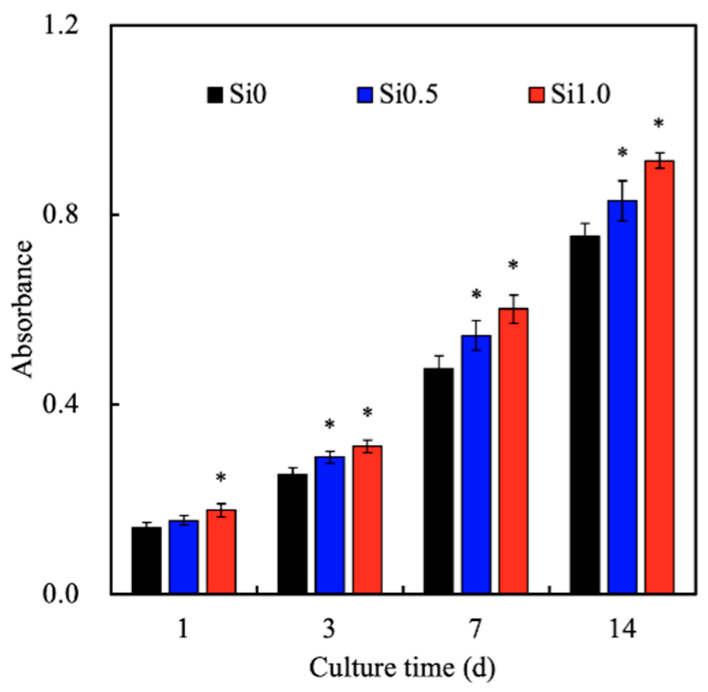
Proliferation rates on the various Si-GelMa hydrogels after different durations of immersion. * indicates significant difference (*p* < 0.05) from Si0.

**Figure 6:**
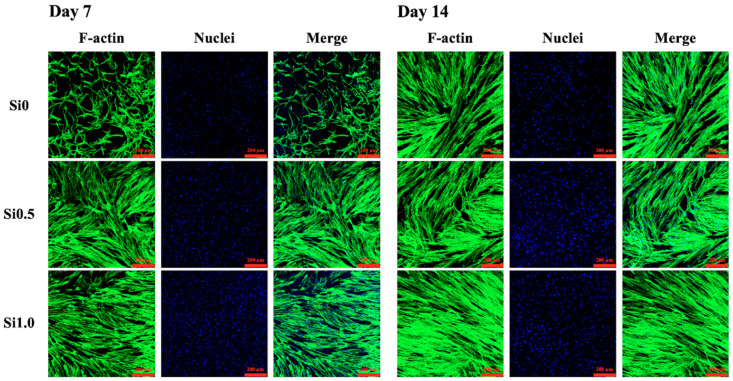
F-actin (green) and nucleus (blue) stain of human dermal fibroblasts (hDF) cultured on various hydrogels for 7 and 14 days. The scale bar is 200 µm.

The proliferation rates of the hDFs cultured on the Si-GelMa hydrogels were assessed and shown in Figure 5. As expected, the incorporation of Si ions enhanced cellular proliferation, and it was found that Si1.0 had significantly higher proliferation after day 1 of culture as compared to the rest of the groups. On day 14 of culturing, Si1.0 had approximately 22% higher proliferation as compared to Si0. Even though Si0.5 had a slightly slower proliferation rate as compared to Si1.0, it still showed a significantly higher proliferation rate from day 3 onwards as compared to Si0. According to Zhang et al., increasing concentrations of hydrogels increases cellular proliferation because the internal architecture of the hydrogels is more compact, thus allowing cells to have better adhesion contact points [35]. There have been various studies supporting the premise that Si ions regulate and stimulate the osteogenic and angiogenic differentiation of stromal cells and endothelial cells by stimulating cellular motility and movement [27,36]. Therefore, incorporating the CS extract into the GelMa hydrogel matrix provides the hydrogel with bioactivity that leads to improved cell migration and angiogenesis due to the release of Si ions [37]. Zhao et al. indicated that Si ions are sufficient to antagonize WNT inhibitors, thus inducing anti-inflammatory responses [38]. Furthermore, stiffer hydrogels have more cell binding sequences, such as the Arg-Gly-Asp RGD motifs, which may promote cellular attachment on the hydrogels. In this study, the results showed the innate biocompatibility of the 3D Si-GelMa hydrogel and the significant stimulation of hDF proliferation in vitro. An important aspect of a good hydrogel for skin tissue engineering is its cytocompatibility. The in vitro cytocompatibility of the hydrogels in the present study was examined through an analysis of the encapsulated cell viability and spreading [39]. As seen from the F-actin and staining results shown in Figure 6, the hDFs in Si1.0 were more well spread as compared to the other groups, thus confirming the role of the Si ions as a cell adhesion promoter [40]. In a recent study, we indicated that the adhesion behavior of hMSCs is regulated by the Si ion concentrations in the substrate affected in part by the identity of ECM ligands initially deposited on the substrate [41]. Furthermore, Si0.5 and Si0 had fewer cell nuclei, thus indicating that Si1.0 was able to promote cellular adhesion and proliferation.

### 3.4. Mitogen-Activated Protein Kinase (MAPK)

**Figure 7:**
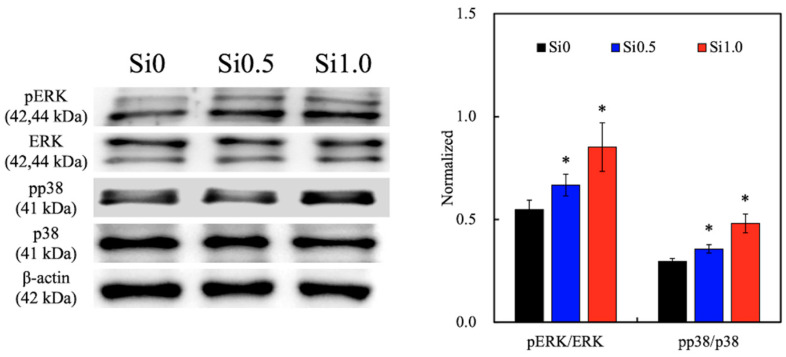
Western blotting and quantification results of phospho-extracellular signal-regulated kinase (pERK), extracellular signal-regulated protein kinases (ERK), pp38 and p38 expression of hDF cultured on Si-GelMa hydrogels for 1 day. “*” indicates a significant difference (*p* < 0.05) compared with Si0.

The levels of several cell signaling-related proteins such as phospho-extracellular signal-regulated protein kinases (pERK), extracellular signal-regulated protein kinases (ERK), p38 mitogen-activated protein kinases (p38), and phosphorylated p38 were evaluated using Western blotting, for which the results are shown in Figure 7. Cells cultured with increasing concentrations of Si ions had darker bands of the various proteins, as seen from the Western blot results. The quantitative results for both pERK/ERK and pp38/p38 showed that Si1.0 and Si0.5 were expressed at significantly higher levels as compared to Si0. These proteins are known to be critical factors in the cellular downstream signaling network that allows cells to carry out their downstream functions, such as the proliferation or secretion of proteins [21]. Huang et al. found that ERK activity is time- and dose-dependent on CS-based material extracts [42]. The current Western blotting results consistently showed that pERK protein is involved in the Si-induced signaling pathway. In addition, p38 can be stimulated in response to growth factors to regulate various differentiation proteins expression in primary cells [43]. p38 has been shown to support cellular responses toward hyperosmotic stress [44]. In a previous study, we proved a common mechanism of p38/MAPK activation in a high osmolality microenvironment, possibly originating from cytoskeletal alterations due to cell shrinkage, as proposed previously in a report involving the Rac protein [45]. Our findings explained that the Si ions released from GelMa hydrogel were induced by osmolality to activate p38 in the hDFs. This series of signaling begins when extracellular stimuli bind with receptor tyrosine kinase or G-protein coupled receptor and trigger the activation of downstream kinases known as MAPKs [46]. Once activated, the MAPKs then set off to phosphorylation-specific substrates that lead to downstream cellular activities. 

### 3.5. Collagen I Secretion

**Figure 8:**
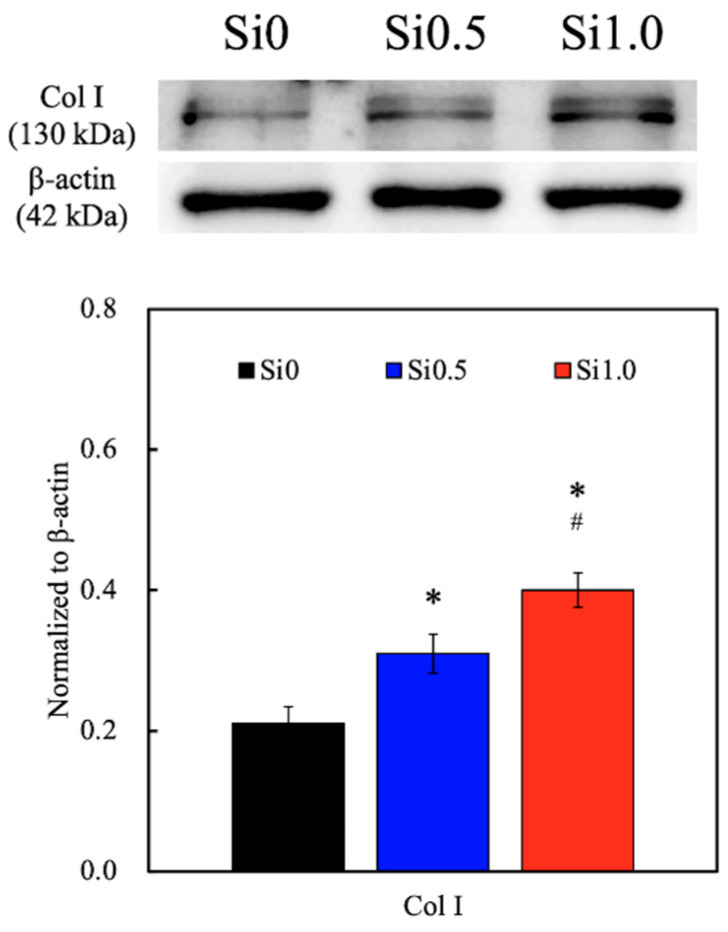
Western blot and quantification results of the Col I synthesis from hDF cultured on Si-GelMa hydrogels for 3 day. “*” indicates a significant difference (*p* < 0.05) compared with Si0. # indicates a significant difference (*p* < 0.05) from Si0.5.

**Figure 9:**
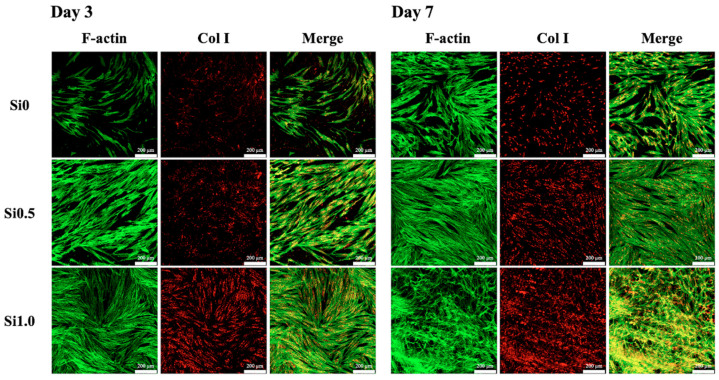
Immunofluorescence staining of Col I expression of hDF cultured on Si-GelMa hydrogels for 3 and 7 days. The scale bar is 200 µm.

Skin is essentially composed of cells (mainly fibroblasts, endothelial cells, and keratinocytes) and ECM. The latter is a dynamic, organized interlocking mesh of many different secreted macromolecules and proteolytic enzymes, where Col I make up almost 90% of the ECM [47]. The main function of Col I is to form the backbone of the skin and to maintain skin structure and tissue integrity. Therefore, Col I can be used as a reliable marker by which to predict subsequent skin tissue regeneration capability. In the present study, the levels of Col I secreted by the hDFs were measured using a Western blot, as shown in Figure 8. As seen from the Western blot results, Si1.0 had darker bands as compared to the rest of the groups, thus indicating that there were higher levels of Col I. The quantification results similarly showed that Si1.0 had significantly higher levels of Col I as compared to Si0.5 and Si0 and that Si0.5 had significantly higher levels of Col I as compared to Si0. To further observe the cell proliferation and function, the samples were stained with F-actin and Col I immunofluorescence stains, for which the results are shown in Figure 9. Several observations were noted after 7 days of culture. Firstly, it could be seen that there were obviously more cells, as seen by the increased amount of staining in the Si1.0 group as compared to Si0 or Si0.5. This result was in agreement with the proliferation results above. In addition, the cells in Si0 were flatter and had longer F-actin spindles as compared to the other groups, thus indicating that the cells were better adhered onto the surfaces of the hydrogel. Previous studies have suggested that Si plays a role in the stimulation of prolyl hydroxylase, which is an enzyme included in collagen assembly [21,22]. Therefore, there were increased levels of Col I in the Si1.0 group as compared to the rest of the groups, thus indicating that Si1.0 was able to enhance skin tissue regeneration. 

### 3.6. Protein Expression of hDF on Si-GelMa Hydrogel

**Figure 10:**
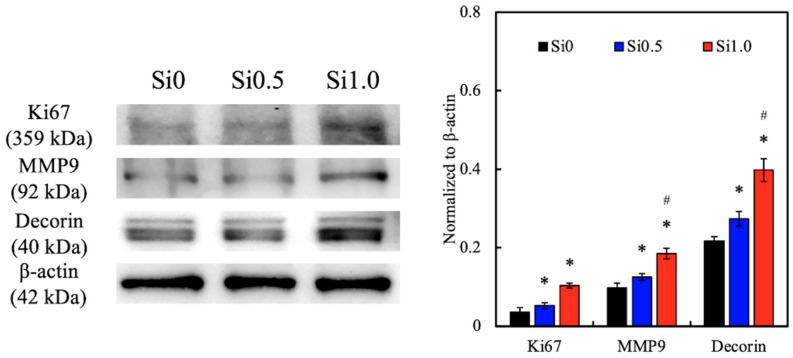
The in vitro effects of the extracellular matrix (ECM) remodeling marker (Ki67, MMP9, and decorin) of hDF cultured on Si-GelMa hydrogels for 14 days. * indicates a significant difference (*p* < 0.05) from Si0. # indicates a significant difference (*p* < 0.05) from Si0.5.

The levels of various biomarkers such as Ki67, MMP9, and Decorin were analyzed using Western blotting and quantification to determine the cellular activities, for which the results are shown in Figure 10. As seen from the Western blot results, Si1.0 had darker bands of Ki67, MMP9, and Decorin, which was further confirmed by the quantification results. Si1.0 and Si0.5 had significantly higher levels of all markers as compared to Si0, and Si1.0 had significant higher levels of MMP9 and Decorin as compared to Si0.5. All three markers were found to be involved in either cell proliferation directly or to acts via the activation of downstream signals. Ki67 is a nuclear protein that is involved in cell proliferation and is also known to be an excellent marker for predicting the growth fraction of a specific cell population [48]. Therefore, it could be seen that Si1.0 was able to enhance cellular proliferation, which was in accordance with the proliferation results discussed above. In addition, MMP9 belongs to a family of proteinases known as matrix metalloproteinases, which are commonly known to be involved in degrading the extracellular matrix and are involved in regulating cell development, especially in cancer evolution and wound regeneration [49]. Matrix metalloproteinases (MMPs) have been reported to be present in both acute and chronic wounds and are critical in regulating the extracellular matrix degradation and deposition that are crucial for wound re-epithelization. However, studies have reported the importance of timing of MMP expression and activation in order to obtain successful wound healing. Thus far, studies involving MMP knockout mice have reported impaired wound healing, where MMP9 is expressed in several types of injured epithelial tissues, including the skin. MMP9 was recently found to play an important role in keratinocyte migration and is expressed at the edge of migrating keratinocytes. On the other hand, decorin is a small leucine-rich proteoglycan that is found in the ECM and is known to regulate remodeling of the ECM, thus leading to scar formation and skin tissue regeneration [50]. Taken together, these results showed that the addition of CS extract may regulate cellular proliferation and ECM remodeling, therefore leading to enhanced skin tissue regeneration. 

## 4. Conclusions

In conclusion, we successfully developed a GelMa hydrogel containing Si ions for wound healing and skin tissue regeneration. The hydrogel had better mechanical properties, which makes it more suitable for surgical handling and implantations. Furthermore, the bioactivity and biocompatibility of the GelMa hydrogel was significantly enhanced by the addition of a CS extract via the release of Ca and Si ions, which was further confirmed by the enhanced cellular adhesion, proliferation, and increased expression of ECM remodeling-related biomarkers, such as Ki67, MMP9, and Decorin. In addition, the release of the ions was shown to increase the adhesiveness of the hydrogels to tissues, thus making them useful for preventing implant dislocation and healing impairment. The enhanced deposition of Col I demonstrated that such a modification could promote in vitro wound healing and thus has the potential to be considered as a clinical application for wound regeneration.

## Data Availability

Data available in a publicly accessible repository.

## References

[1] Lin Y.H., Chuang T.Y., Chiang W.H., Chen I.W.P., Wang K., Shie M.Y., Chen Y.W. (2019). The synergistic effects of graphene-contained 3D-printed calcium silicate/poly-ε-caprolactone scaffolds promote FGFR-induced osteogenic/angiogenic differentiation of mesenchymal stem cells. Mater. Sci. Eng. C Mater. Biol. Appl..

[2] Elomaa L., Keshi E., Sauer I.M., Weinhart M. (2020). Development of GelMA/PCL and dECM/PCL resins for 3D printing of acellular in vitro tissue scaffolds by stereolithography. Mater. Sci. Eng. C Mater. Biol. Appl..

[3] Hussein K.H., Abdelhamid H.N., Zou X., Woo H.-M. (2019). Ultrasonicated graphene oxide enhances bone and skin wound regeneration. Mater. Sci. Eng. C Mater. Biol. Appl..

[4] Shefa A.A., Sultana T., Park M.K., Lee S.Y., Gwon J.-G., Lee B.T. (2020). Curcumin incorporation into an oxidized cellulose nanofiber-polyvinyl alcohol hydrogel system promotes wound healing. Mater. Des..

[5] Sundarakrishnan A., Zukas H., Coburn J., Bertini B.T., Liu Z., Georgakoudi I., Baugh L., Dasgupta Q., Black L.D., Kaplan D.L. (2019). Bioengineered in vitro tissue model of fibroblast activation for modeling pulmonary fibrosis. ACS Biomater. Sci. Eng..

[6] Chong L.H., Lim M.M., Sultana N. (2015). Fabrication and evaluation of polycaprolactone/gelatin-based electrospun nanofibers with antibacterial properties. J. Nanomater..

[7] Mao Q., Hoffmann O., Yu K., Lu F., Lan G., Dai F., Shang S., Xie R. (2020). Self-contracting oxidized starch/gelatin hydrogel for noninvasive wound closure and wound healing. Mater. Des..

[8] Thangprasert A., Tansakul C., Thuaksubun N., Meesane J. (2019). Mimicked hybrid hydrogel based on gelatin/PVA for tissue engineering in subchondral bone interface for osteoarthritis surgery. Mater. Des..

[9] Hao Y., Zhao W., Zhang L., Zeng X., Sun Z., Zhang D., Shen P., Li Z., Han Y., Li P. (2020). Bio-multifunctional alginate/chitosan/fucoidan sponges with enhanced angiogenesis and hair follicle regeneration for promoting full-thickness wound healing. Mater. Des..

[10] Wu G., Ma X., Fan L., Gao Y., Deng H., Wang Y. (2020). Accelerating dermal wound healing and mitigating excessive scar formation using LBL modified nanofibrous mats. Mater. Des..

[11] Wang Y., Zhang R., Qin W., Dai J., Zhang Q., Lee K., Liu Y. (2020). Physicochemical properties of gelatin films containing tea polyphenol-loaded chitosan nanoparticles generated by electrospray. Mater. Des..

[12] Liu J., Li L., Suo H., Yan M., Yin J., Fu J. (2019). 3D printing of biomimetic multi-layered GelMA/nHA scaffold for osteochondral defect repair. Mater. Des..

[13] Juncos Bombin A.D., Dunne N.J., McCarthy H.O. (2020). Electrospinning of natural polymers for the production of nanofibres for wound healing applications. Mater. Sci. Eng. C Mater. Biol. Appl..

[14] Afjoul H., Shamloo A., Kamali A. (2020). Freeze-gelled alginate/gelatin scaffolds for wound healing applications: An in vitro, in vivo study. Mater. Sci. Eng. C Mater. Biol. Appl..

[15] Shie M.Y., Lee J.J., Ho C.C., Yen S.Y., Ng H.Y., Chen Y.W. (2020). Effects of gelatin methacrylate bio-ink concentration on mechano-physical properties and human dermal fibroblast behavior. Polymers.

[16] Chen M., Zhao F., Li Y., Wang M., Chen X., Lei B. (2020). 3D-printed photoluminescent bioactive scaffolds with biomimetic elastomeric surface for enhanced bone tissue engineering. Mater. Sci. Eng. C Mater. Biol. Appl..

[17] Zou Q., Grottkau B.E., He Z., Shu L., Yang L., Ma M., Ye C. (2020). Biofabrication of valentine-shaped heart with a composite hydrogel and sacrificial material. Mater. Sci. Eng. C Mater. Biol. Appl..

[18] Bedir T., Ulag S., Ustundag C.B., Gunduz O. (2020). 3D bioprinting applications in neural tissue engineering for spinal cord injury repair. Mater. Sci. Eng. C Mater. Biol. Appl..

[19] Le Duigou A., Correa D., Ueda M., Matsuzaki R., Castro M. (2020). A review of 3D and 4D printing of natural fibre biocomposites. Mater. Des..

[20] Chen Q., Wu J., Liu Y., Li Y., Zhang C., Qi W., Yeung K.W.K., Wong T.M., Zhao X., Pan H. (2019). Electrospun chitosan/PVA/bioglass Nanofibrous membrane with spatially designed structure for accelerating chronic wound healing. Mater. Sci. Eng. C Mater. Biol. Appl..

[21] Shie M.Y., Ding S.J. (2013). Integrin binding and MAPK signal pathways in primary cell responses to surface chemistry of calcium silicate cements. Biomaterials.

[22] Shie M.Y., Ding S.J., Chang H.C. (2011). The role of silicon in osteoblast-like cell proliferation and apoptosis. Acta Biomater..

[23] Tu M.G., Lee K.X., Lin Y.H., Huang T.H., Ho C.C., Shie M.Y. (2020). Caffeic acid-coated nanolayer on Mineral Trioxide Aggregate potentiate the host immune responses, angiogenesis, and odontogenesis. J. Endod..

[24] Chen Y.W., Shen Y.F., Ho C.C., Yu J., Wu Y.H., Wang K., Shih C.T., Shie M.Y. (2018). Osteogenic and angiogenic potentials of the cell-laden hydrogel/mussel-inspired calcium silicate complex hierarchical porous scaffold fabricated by 3D bioprinting. Mater. Sci. Eng. C Mater. Biol. Appl..

[25] Wang X., Chang J., Wu C. (2018). Bioactive inorganic/organic nanocomposites for wound healing. Appl. Mater. Today.

[26] Gao L., Zhou Y., Peng J., Xu C., Xu Q., Xing M., Chang J. (2019). A novel dual-adhesive and bioactive hydrogel activated by bioglass for wound healing. NPG Asia Mater..

[27] Chiu Y.C., Shie M.Y., Lin Y.H., Lee K.X., Chen Y.W. (2019). Effect of strontium substitution on the physicochemical properties and bone regeneration potential of 3D printed calcium silicate scaffolds. Int. J. Mol. Sci..

[28] Shaker M.A., Doré J.J.E., Younes H.M. (2010). Synthesis, characterization and cytocompatibility of a poly(diol-tricarballylate) visible light photo-cross-linked biodegradable elastomer. J. Biomat. Sci. Polym. E.

[29] Sujan M.I., Sarkar S.D., Sultana S., Bushra L., Tareq R., Roy C.K., Azam M.S. (2020). Bi-functional silica nanoparticles for simultaneous enhancement of mechanical strength and swelling capacity of hydrogels. RSC Adv..

[30] Liu Y., Pharr M., Salvatore G.A. (2017). Lab-on-skin: A review of flexible and stretchable electronics for wearable health monitoring. ACS Nano.

[31] Trappmann B., Gautrot J.E., Connelly J.T., Strange D.G.T., Li Y., Oyen M.L., Cohen Stuart M.A., Boehm H., Li B., Vogel V. (2012). Extracellular-matrix tethering regulates stem-cell fate. Nat. Mater..

[32] Bittner S.M., Smith B.T., Diaz-Gomez L., Hudgins C.D., Melchiorri A.J., Scott D.W., Fisher J.P., Mikos A.G. (2019). Fabrication and mechanical characterization of 3D printed vertical uniform and gradient scaffolds for bone and osteochondral tissue engineering. Acta Biomater..

[33] Chen Y.W., Hsu T.T., Wang K., Shie M.Y. (2016). Preparation of the fast setting and degrading Ca-Si-Mg cement with both odontogenesis and angiogenesis differentiation of human periodontal ligament cells. Mater. Sci. Eng. C Mater. Biol. Appl..

[34] Yu C.T., Wang F.M., Liu Y.T., Ng H.Y., Jhong Y.R., Hung C.H., Chen Y.W. (2020). Effect of bone morphogenic protein-2 loaded mesoporous strontium substitution calcium silicate/recycled fish gelatin 3D cell-laden scaffold for bone tissue engineering. Processes.

[35] Zhang J., Yun S., Du Y., Zannettino A., Zhang H. (2020). Hydrogel-based preparation of cell aggregates for biomedical applications. Appl. Mater. Today.

[36] Gandolfi M.G., Iezzi G., Piattelli A., Prati C., Scarano A. (2017). Osteoinductive potential and bone-bonding ability of ProRoot MTA, MTA Plus and Biodentine in rabbit intramedullary model: Microchemical characterization and histological analysis. Dent. Mater..

[37] Yu H., Peng J., Xu Y., Chang J., Li H. (2016). Bioglass activated skin tissue engineering constructs for wound healing. ACS Appl. Mater. Interfaces.

[38] Zhao Y., Yuan X., Bellido T., Helms J.A. (2019). A correlation between Wnt/beta-catenin signaling and the rate of dentin secretion. J. Endod..

[39] Chen Y.W., Wang K., Ho C.C., Kao C.T., Ng H.Y., Shie M.Y. (2020). Cyclic tensile stimulation enrichment of Schwann cell-laden auxetic hydrogel scaffolds towards peripheral nerve tissue engineering. Mater. Des..

[40] Kao C.T., Chiu Y.C., Lee K.X., Lin Y.H., Huang T.H., Liu Y.C., Shie M.Y. (2021). The synergistic effects of Xu Duan combined Sr-contained calcium silicate/poly-ε-caprolactone scaffolds for the promotion of osteogenesis marker expression and the induction of bone regeneration in osteoporosis. Mater. Sci. Eng. C Mater. Biol. Appl..

[41] Shie M.Y., Chang H.C., Ding S.J. (2014). Composition-dependent protein secretion and integrin level of osteoblastic cell on calcium silicate cements. J. Biomed. Mater. Res. Part A.

[42] Huang T.H., Ding S.J., Hsu T.C., Kao C.T. (2003). Effects of mineral trioxide aggregate (MTA) extracts on mitogen-activated protein kinase activity in human osteosarcoma cell line (U2OS). Biomaterials.

[43] Chiu Y.C., Fang H.Y., Hsu T.T., Lin C.Y., Shie M.Y. (2017). The characteristics of Mineral Trioxide Aggregate/polycaprolactone 3-dimensional scaffold with osteogenesis properties for tissue regeneration. J. Endod..

[44] Mavrogonatou E., Kletsas D. (2011). Differential response of nucleus pulposus intervertebral disc cells to high salt, sorbitol, and urea. J. Cell. Physiol..

[45] Uhlik M.T., Abell A.N., Johnson N.L., Sun W., Cuevas B.D., Lobel-Rice K.E., Horne E.A., Dell’Acqua M.L., Johnson G.L. (2003). Rac–MEKK3–MKK3 scaffolding for p38 MAPK activation during hyperosmotic shock. Nat. Cell Biol..

[46] Ma H., Feng C., Chang J., Wu C. (2018). 3D-printed bioceramic scaffolds: From bone tissue engineering to tumor therapy. Acta Biomater..

[47] Huang S., Fu X. (2010). Naturally derived materials-based cell and drug delivery systems in skin regeneration. J. Control. Release.

[48] Shen J., Ji Y., Xie M., Zhao H., Xuan W., Yin L., Yu X., Xu F., Su S., Nie J. (2020). Cell-modified bioprinted microspheres for vascular regeneration. Mater. Sci. Eng. C Mater. Biol. Appl..

[49] Lazurko C., Khatoon Z., Goel K., Sedlakova V., Eren Cimenci C., Ahumada M., Zhang L., Mah T.F., Franco W., Suuronen E.J. (2019). Multifunctional nano and collagen-based therapeutic materials for skin repair. ACS Biomater. Sci. Eng..

[50] Abalymov A., Parakhonskiy B., Skirtach A.G. (2020). Polymer- and hybrid-based biomaterials for interstitial, connective, vascular, nerve, visceral and musculoskeletal tissue engineering. Polymers.

